# Cognitive reserve and effects of air pollution mixture on cognitive function in dementia-free adults

**DOI:** 10.1017/S0033291726103407

**Published:** 2026-02-02

**Authors:** Juyeon Ko, Young Noh, Sang-Baek Koh, Seung-Koo Lee, Sun-Young Kim, Hong-Nguyen Tran-Thi, Jaelim Cho, Changsoo Kim

**Affiliations:** 1 Department of Preventive Medicine, Yonsei University College of Medicine, Seoul, Republic of Korea; 2 Department of Neurology, Gil Medical Centre, Gachon University College of Medicine, Incheon, Republic of Korea; 3 Department of Preventive Medicine, Wonju College of Medicine, Yonsei University, Wonju, Republic of Korea; 4 Department of Cancer Control and Population Health, Graduate School of Cancer Science and Policy, National Cancer Centre, Goyang, Republic of Korea; 5 University of Medicine and Pharmacy, Ho Chi Minh City, Vietnam; 6 Institute for Environmental Research, Yonsei University College of Medicine, Seoul, Republic of Korea; 7 Institute of Human Complexity and Systems Science, Yonsei University, Incheon, Republic of Korea

**Keywords:** air pollution, brain health, cognitive impairment, cognitive reserve, MoCA score

## Abstract

**Background:**

Extensive evidence links air pollution exposure to cognitive decline; however, it remains unclear whether cognitive reserve and brain reserve modify this association. We examined the moderating roles of cognitive reserve contributors and brain reserve in the association between air pollution and cognitive function in dementia-free adults.

**Methods:**

Cross-sectional data were obtained from 650 participants who underwent 3T brain magnetic resonance imaging and completed the Montreal Cognitive Assessment (MoCA). Cognitive reserve contributors were assessed based on education, occupation, and social engagement. Brain reserve was quantified using the ventricle-to-brain ratio derived from brain scans. Five-year average concentrations of particulate matter with diameters ≤10 and ≤2.5 μm and nitrogen dioxide were estimated based on residential addresses. Partial least squares structural equation modeling was applied to construct latent variables representing the air pollution mixture and composite cognitive reserve (contributors). Analyses examined whether cognitive reserve contributors and brain reserve modified associations of air pollution with MoCA scores and suspected mild cognitive impairment.

**Results:**

In individuals with an average level of cognitive reserve, a 1–standard deviation increase in air pollution mixture was associated with a 0.24-point decrease in MoCA scores (95% confidence interval [CI]: −0.31 to −0.16). This association was attenuated in individuals with higher cognitive reserve (*β* = −0.12; 95% CI: −0.25 to 0.02) and intensified in those with lower cognitive reserve (*β* = −0.36; 95% CI: −0.37 to −0.35). The moderating effect of brain reserve was not significant.

**Conclusions:**

Higher cognitive reserve may mitigate the effects of air pollution on cognitive function.

## Introduction

Neurodegenerative disorders, such as Alzheimer’s disease and mild cognitive impairment (MCI), are major public health concerns. These conditions are typically characterized by cognitive decline; however, some individuals exhibit fewer clinical symptoms, despite having similar levels of brain pathology (Neuropathology Group & Medical Research Council Cognitive Function and Aging Study, 2001). This discrepancy can be explained by the concepts of cognitive and brain reserves. Brain reserve refers to passive and structural aspects of the brain, such as brain size and number of neurons, which may help individuals tolerate neurodegeneration (Stern et al., 2020). By contrast, cognitive reserve is an active form of reserve that reflects the brain’s ability to cope with pathology through the flexible and efficient use of cognitive processes (Stern et al., 2020). Although cognitive reserve manifests its protective effects later in life, it is shaped earlier through cumulative life experiences, including education, occupation, and social engagement, which are commonly referred to as cognitive reserve contributors (Opdebeeck, Martyr, & Clare, 2016; Stern et al., 2020). Furthermore, recent studies have shown that a composite measure of cognitive reserve contributors, which integrates education, occupation, and social engagement, is more strongly associated with cognitive function in older adults than any single contributor alone (Khalaila, Dintica, & Yaffe, 2024; Yang et al., 2024).

Epidemiological studies have demonstrated that exposure to particulate matter (PM) increases the risk of neurodegenerative disorders, including Alzheimer’s disease (Fu, Guo, Cheung, & Yung, 2019; Gong et al., 2023; Tsai et al., 2019). Large population-based studies have also shown that exposure to air pollution is associated with poorer cognitive function in individuals without dementia (Duchesne et al., 2022; Kulick et al., 2020; Wang et al., 2020). Neuroimaging studies have supported these findings by revealing associations between PM exposure and reductions in cortical volume and thickness (subclinical markers of Alzheimer’s disease) (Gale et al., 2020; Ko et al., 2024). Similar associations were observed in the case of nitrogen dioxide (NO_2_) exposure (Cho et al., 2020; Gale et al., 2020). These effects are thought to involve systemic inflammation, oxidative stress, and neurovascular injury (Calderón-Garcidueñas et al., 2004). However, most studies have focused on single-pollutant models and did not consider the combined effects of multiple pollutants on cognitive function.

Although cognitive and brain reserves are known to benefit cognitive health, their moderating effects on the relationship between air pollution and cognitive function remain unclear. Several studies have shown that education mitigates the effect of air pollution on cognitive function; individuals with lower educational attainment experience greater cognitive decline due to air pollution exposure (PM with aerodynamic diameters ≤2.5 μm [PM_2.5_], PM with aerodynamic diameters ≤10 μm [PM_10_], and NO_2_), whereas those with higher education are less affected (Ailshire & Walsemann, 2021; Tan et al., 2021; Yao, Wang, & Xiang, 2022; Zhang, Chen, & Zhang, 2018). However, findings on social engagement are inconsistent, with some studies reporting a protective effect (Frndak et al., 2024; Tan et al., 2021) and others showing no significant association (Ilango et al., 2023; Tallon et al., 2017). To date, most epidemiological evidence has focused on individual cognitive reserve contributors, such as education or social engagement, rather than a composite measure that captures cumulative cognitive enrichment across the life course. Yet, these proxies capture only one facet of lifelong cognitive enrichment. A composite measure of cognitive reserve contributors that integrates education, occupation, and social engagement provides a more comprehensive representation of cumulative reserve across cognitive, occupational, and social domains (Khalaila et al., 2024; Yang et al., 2024). Moreover, the role of brain reserve in moderating the effects of pollution mixtures has rarely been explored.

The present study aimed to investigate the moderating effects of cognitive and brain reserves on the association between exposure to an air pollution mixture and cognitive function.

## Materials and methods

### Study population

**Figure 1.**
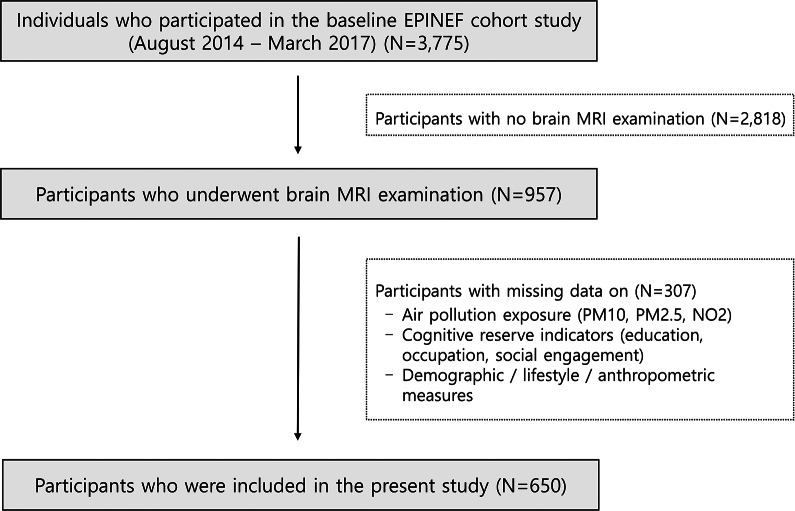
Flowchart of participant selection in the Environmental Pollution-Induced Neurological EFfects (EPINEF) cohort study. *Note*: Participants with a history of individuals with neurological diseases (dementia, stroke, and Parkinson’s disease) were excluded.

This study used baseline cross-sectional data from the Environmental Pollution-Induced Neurological EFfects cohort study (Jang et al., 2021). Participants were recruited via local advertisements from four cities in the Republic of Korea: Seoul, Incheon, Wonju, and PyeongChang. Individuals with a history of neurological diseases, including dementia, stroke, or Parkinson’s disease, were excluded. Among individuals who participated in the baseline survey conducted between August 2014 and March 2017 at three university hospitals, 957 participants underwent brain magnetic resonance imaging (MRI) using either a Philips 3T Achieva or a Siemens 3T Verio scanner. Cognitive function was assessed using the Korean version of the Montreal Cognitive Assessment (MoCA) among the participants who underwent brain MRI scanning with a Philips 3T Achieva scanner. A total of 650 participants with complete data on air pollution exposure (PM_10_, PM_2.5_, and NO_2_), cognitive reserve contributor variables, brain MRI, MoCA score, demographic characteristics, lifestyle habits, disease history, and anthropometric measures were included in the analysis (Figure 1).

Written informed consent was obtained from all participants, and the study was approved by the Yonsei University Health System Institutional Review Board (approval number 4–2022-0418).

### Exposure assessment

Individual long-term exposures to PM_10_ and NO_2_ were assessed using 5-year average concentrations estimated at each participant’s residential address before the enrollment year. The concentrations were estimated using universal kriging model based on regulatory air quality monitoring data from ~300 sites between 2001 and 2016 (Kim & Song, 2017). For PM_2.5_, the 1-year average concentration in 2015 was estimated at each participant’s residential address because national monitoring data for PM_2.5_ have been available only since 2015 in the Republic of Korea.

### Cognitive reserve contributors

Information on cognitive reserve contributors, defined as measurable life-experience factors, including education level, occupation, and social engagement, was collected at baseline using structured questionnaires. The questionnaires were adapted from previous studies and revised as needed (Nucci, Mapelli, & Mondini, 2012; Yang et al., 2024).

Education was defined as the number of years of formal education. Occupation was determined based on participants’ employment status and job titles, which were then matched to job codes from the Standard Occupational Classification 2000 system of the UK Office for National Statistics (Office for National Statistics, 2022). The Standard Occupational Classification provides a hierarchical classification of occupations according to skill level and specialization, ranging from managers and professional occupations to elementary occupations (Ko et al., 2022; Office for National Statistics, 2022). These job codes were converted into the Socioeconomic Classification (SEC), an ordinal variable ranging from 1.1 and 1.2 to 7, with lower values indicating higher occupational status and skill levels (Ko et al., 2022; Office for National Statistics, 2022). Occupational attainment was then recoded into four levels: (1) unemployed, looking after home and/or family, unable to work due to illness or disability, or classified as SEC 5–7; (2) SEC 3 or SEC 4; (3) SEC 2; and (4) SEC 1.1 or SEC 1.2.

Social engagement was assessed by asking the participants whether they engaged in specific activities at least once a month. These activities included religious activities, social gatherings (i.e., alumni meetings and family reunions), sports or gymnasium involvement, and volunteer work. Each activity type was recorded as a “yes” or “no.” Participants could report “yes” for multiple activities, indicating engagement in more than one type of social activity.

### Estimation of brain reserve

Three-dimensional T1-magnetization prepared rapid gradient-echo images were obtained using 3T brain MRI. During the MRI scan, the participants were placed in the supine position, and the following imaging parameters were used: repetition time, 1,900 ms; echo time, 2.93 ms; flip angle, 8°; pixel bandwidth, 170 Hz/pixel; matrix size, 256 × 208; field of view, 256 mm; and the number of excitations, 1. FreeSurfer (version 6.0.0; http://surfer.nmr.mgh.harvard.edu/), a standard software tool for the precise analysis of neuroimaging data (Na et al., 2024), was used to estimate the total gray matter (GM), total cerebral white matter (WM), and cerebrospinal fluid (CSF) volume. Brain reserve was quantified using the ventricle-to-brain ratio (VBR). To calculate the VBR, the following equation was applied using the three volumetric indices: GM, WM, and CSF (Bigler et al., 2004).

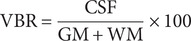

VBR was chosen as the index of brain reserve because ventricular enlargement inversely reflects overall brain volume and neuronal loss, thereby serving as a sensitive measure of current structural reserve status (Apostolova et al., 2012).

### Assessment of cognitive function

We assessed global cognitive function using the Korean version of the MoCA. The MoCA was a brief cognitive screening tool with high sensitivity for screening patients with suspected MCI. The MoCA included the cognitive functions of orientation, attention, language, abstraction, visuospatial/executive function, naming, and episodic memory. The highest possible score on the MoCA was 30, with higher scores indicating better cognitive performance. Suspected MCI was defined as a MoCA score of ≤19 (Lee et al., 2008). The sensitivity, specificity, positive predictive value, and negative predictive value of this cutoff were 51%, 97%, 83%, and 86%, respectively, for discriminating suspected MCI (Lee et al., 2008).

### Covariates

At baseline, information on demographic characteristics (age and sex), lifestyle habits (alcohol consumption and smoking status), history of diseases (diabetes and hypertension), as well as anthropometric measures (height, weight and body mass index) was collected. Smoking status was categorized as ever-smoker and never-smoker. Alcohol consumption was categorized as a current drinker or non-drinker. The body mass index (kg/m^2^) was calculated as weight (kg) divided by the square of height (m).

Blood samples were obtained following a minimum 12-h fasting period and subsequently analyzed at a central laboratory (Seoul Clinical Laboratory Co., Ltd, Seoul, Korea). Hypertension was defined as a systolic blood pressure ≥140 mmHg, a diastolic blood pressure ≥ 90 mmHg, or a diagnosis of hypertension by a physician. Diabetes was defined as a fasting blood glucose level ≥126 mg/dL or a diagnosis of diabetes by a physician.

### Statistical analysis

The results of the analysis of the general characteristics of the study participants were reported as means (standard deviation [SD]), medians (25th to 75th percentiles), or frequencies (%). Linear regression analyses were used to investigate the association between exposure to a single air pollutant (PM_10_, PM_2.5_, or NO_2_) and MoCA scores. The results were reported as beta coefficient (per 10 μg/m^3^ increase in PM_10_ and PM_2.5_ or per 10-ppb increase in NO_2_) along with 95% confidence intervals (95% CIs). The normality of residuals from the linear regression models was assessed using visual inspection of residual QQ plots. No major deviation from normality was observed.

**Figure 2.**
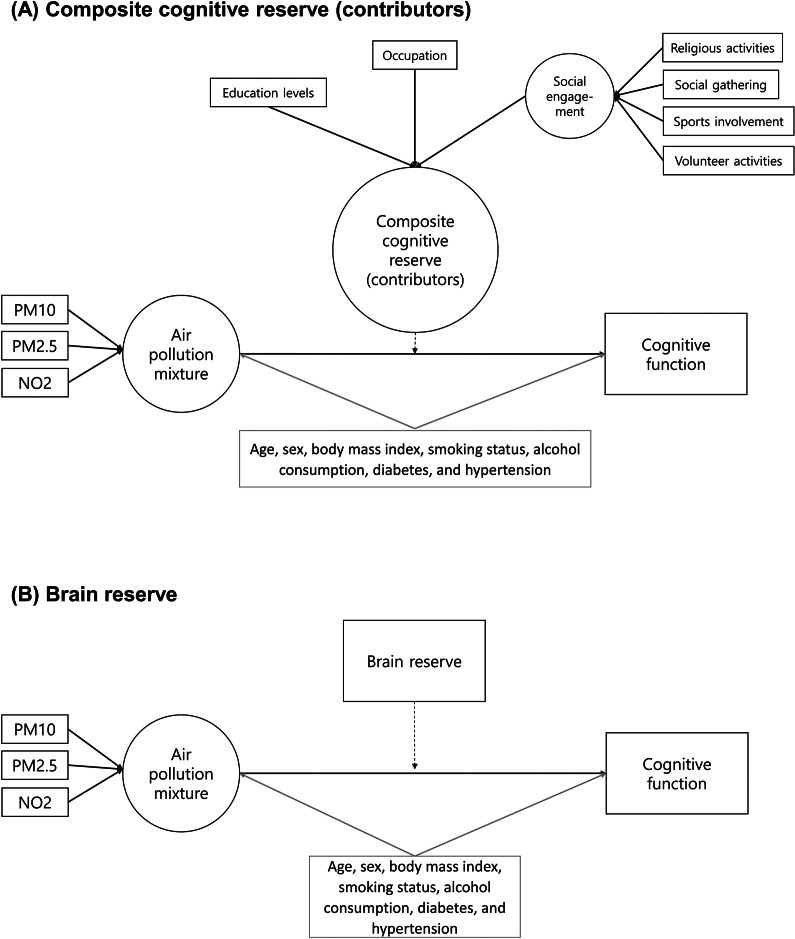
Structural model derived by partial least squares structural equation modeling performed for the moderation analysis of the association between the air pollution mixture and cognitive function. *Abbreviations:* MoCA, Montreal Cognitive Assessment. *Note*: Rectangles indicate observed variables, and ellipses represent latent variables or constructs. Cognitive function variables were the MoCA score, and suspected mild cognitive impairment, defined as a MoCA score ≤ 19. Brain reserve was quantified using the ventricle-to-brain ratio, calculated as the cerebrospinal fluid volume divided by the sum of the total gray matter volume and total cerebral white matter volume, multiplied by 100. Brain reserve values were log-transformed to normalize the distribution.

We used partial least squares structural equation modeling (PLS-SEM) to investigate the moderating effects of cognitive and brain reserve on the association between the air pollution mixture and cognitive outcomes (MoCA score and suspected MCI) (Figure 2A) (Hair et al., 2019). PLS-SEM was selected because of its flexibility in handling complex models without strict normality assumptions and its suitability for formative measurement models. In addition, PLS-SEM internally standardizes variables measured on different scales when estimating latent composites, eliminating the need for external standardization. The air pollution mixture (PM10, PM2.5, and NO2) was modeled as a latent composite using a formative measurement approach. This approach was supported by the weak-to-moderate correlations among PM10, PM2.5, and NO2 concentrations (Pearson’s r values ranging from 0.35 to 0.64, all statistically significant), indicating the importance of accounting for their combined effects rather than evaluating individual pollutants separately. Similarly, we constructed a composite cognitive reserve (contributors) by integrating education, occupation, and a latent social engagement variable. Education (categorized into seven levels based on years of schooling) showed significant associations with occupation and individual social engagement components (chi-squared tests; all *P* < 0.05). The four social engagement factors (i.e., religion, relationships, leisure, and charity work) were first combined into a latent social engagement variable, which was then integrated with education and occupation to form the composite cognitive reserve (all *P* < 0.05). The moderating effects of the composite cognitive reserve and its individual contributors (i.e., education, occupation, and social engagement) were examined separately, alongside brain reserve (Figure 2B). Brain reserve was log-transformed due to its skewed distribution. All models were adjusted for age, sex, body mass index, smoking status, alcohol consumption, diabetes, and hypertension (Giordano et al., 2012; Song, Stern, & Gu, 2022; Zijlmans et al., 2022). Moderating effects were estimated using path coefficients (*β*) and 95% CIs derived from a 5,000-sample bootstrapping procedure. Model quality in the PLS-SEM analysis was evaluated using the coefficient of determination (*R* ^2^) and variance inflation factor (VIF) (Chin, 2001; Chin, Marcolin, & Newsted, 2003; Hair et al., 2016). The *R* ^2^ values were used to assess the proportion of variance explained in the endogenous constructs and were categorized as small (0.02 ≤ *R* ^2^ < 0.13), medium (0.13 ≤ *R* ^2^ < 0.26), or large (*R* ^2^ ≥ 0.26) (Chin et al., 2003). The interaction between the air pollution mixture and the composite cognitive reserve (contributors) or brain reserve explained 13–43% of the variance in MoCA scores and suspected MCI (Supplementary Table 1). Multicollinearity was assessed using VIF, with all values below 5, indicating no significant multicollinearity (Supplementary Table 1) (Giordano et al., 2012; Hair et al., 2019; Song et al., 2022; Zijlmans et al., 2022). We visualized the interaction effects using simple slopes, which were linear lines regressing the MoCA score or suspected MCI on the air pollution mixture at average level (mean), higher (+1 SD), and lower (−1 SD) levels of the composite cognitive reserve (contributors).

All statistical analyses were conducted using SAS software (version 9.4; SAS Institute, Cary, NC, USA), and the PLS-SEM analysis was performed using SmartPLS (version 4). Results were considered statistically significant with two-sided *P*-values <0.05.

## Results

### Characteristics of study participants

The mean age was 67.8 (SD, 6.9) years (Table 1). Men comprised 45.9% of the study population. The mean concentrations of PM_10_, PM_2.5_, and NO_2_ were 49.7 μg/m^3^, 25.9 μg/m^3^, and 27.0 ppb, respectively. The proportion of suspected MCI was 14.6%.

**Table 1.** Characteristics of study participants

Characteristics	Total (*N* = 650)
**Age (years), mean (SD)**	67.8 (6.9)
**Men, *N* (%)**	298 (45.9)
**Body mass index (kg/m** ^**2**^**), mean (SD)**	24.6 (2.9)
**Smoking status, *N* (%)**	
Ever-smoker	223 (34.3)
Never-smoker	427 (65.6)
**Alcohol consumption, *N* (%)**	
Current drinker	269 (41.4)
Non-drinker	381 (58.6)
**Medical history, *N* (%)**	
Diabetes mellitus	146 (22.5)
Hypertension	312 (48.0)
**Ambient air pollutants, mean (SD)**	
PM_10_, μg/m^3^	49.7 (5.2)
PM_2.5_, μg/m^3^	25.9 (0.7)
NO_2_, ppb	27.0 (11.1)
**Cognitive reserve contributors**	
Education levels (years), mean (SD)	9.9 (4.3)
Occupation, *N* (%)	
Unemployed, looking after home and/or family, unable to work due to illness or disability, or classified as SEC 5–7	488 (75.1)
SEC 3 or SEC 4	111 (17.1)
SEC 2	12 (1.9)
SEC 1.1 or SEC 1.2	29 (6.0)
Social engagement, *N* (%)	
Religious activities	367 (56.5)
Social gatherings	494 (76.0)
Sports club or gym participation	371 (57.1)
Volunteer activities	152 (23.4)
**Ventricle-to-brain ratio, median (25%–75%)**	0.12 (0.10–0.14)
**Cognitive test (MoCA) score, mean (SD)**	22.9 (4.4)
**Suspected mild cognitive impairment, *N (%)***	95 (14.6)

*Note*: Long-term exposures to PM_10_ and NO_2_ were defined as 5-year averages of concentrations. For PM_2.5_, a 1-year average concentration was used. Brain reserve was quantified using the ventricle-to-brain ratio, calculated as cerebrospinal fluid divided by the sum of total gray matter volume and total cerebral white matter, multiplied by 100. Suspected mild cognitive impairment was defined as a MoCA score ≤19. Social engagement was assessed using multiple yes/no items.

*Abbreviations:* SD, standard deviation; PM_10_, particulate matter, aerodynamic diameter ≤ 10 μm; PM_2.5_, particulate matter, aerodynamic diameter ≤ 2.5 μm; NO_2_, nitrogen dioxide; SEC, socioeconomic classification; MoCA, Montreal Cognitive Assessment.

### Moderating effects of cognitive reserve contributors and brain reserve on air pollution-associated MoCA score

In the linear regression model, a 10 μg/m^3^ increase in PM_10_ (*β* = −1.09; 95% CI: −1.62 to −0.56), a 10 μg/m^3^ increase in PM_2.5_ (*β* = −4.39; 95% CI: −8.04 to −0.74), and a 10-ppb increase in NO_2_ (*β* = −1.09; 95% CI: −1.38 to −0.80) were significantly associated with lower MoCA scores.

**Figure 3.**
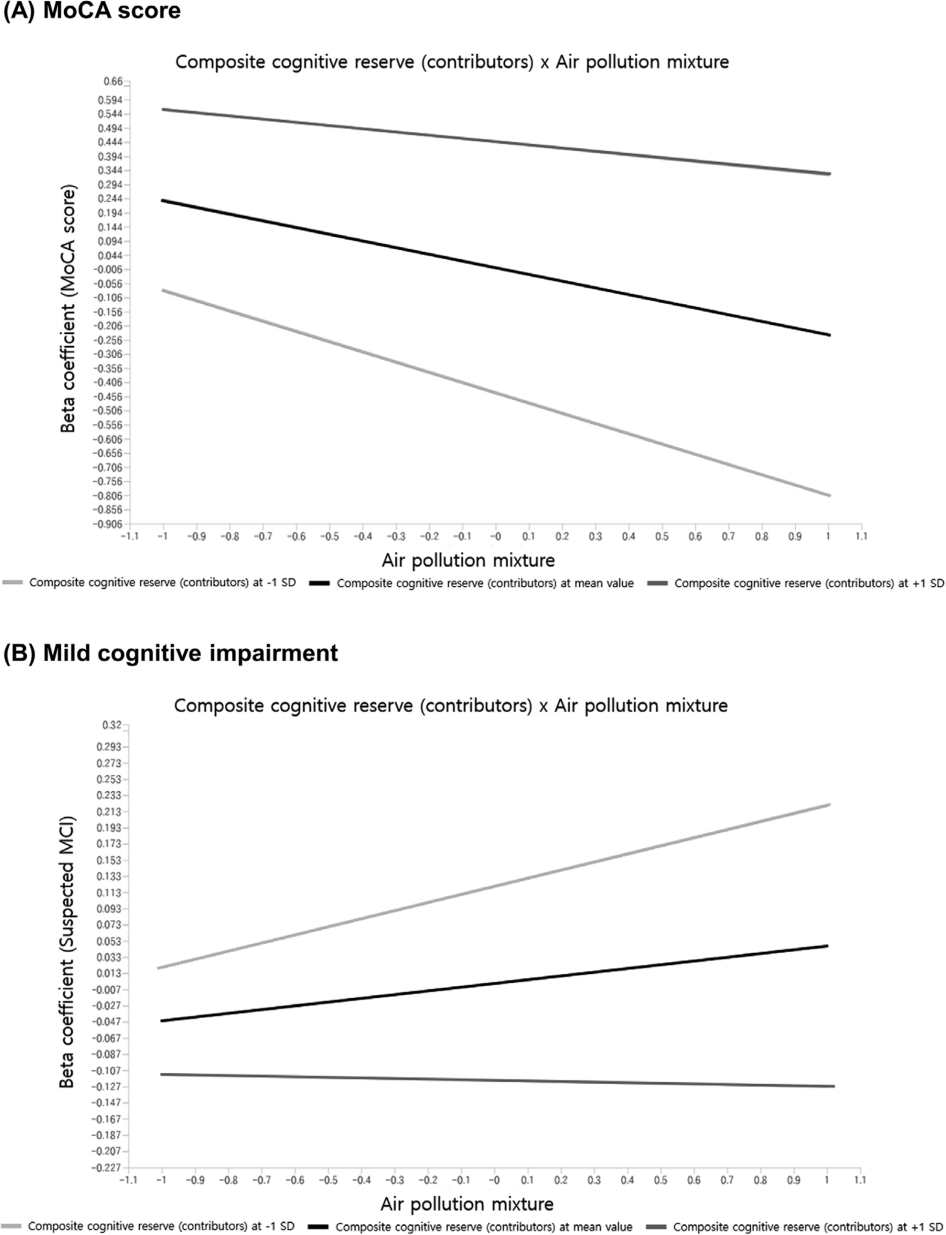
Simple slope plots showing the associations between the air pollution mixture and MoCA score (A) or mild cognitive impairment (B), moderated by the composite cognitive reserve (contributors). *Abbreviations:* MoCA, Montreal Cognitive Assessment; MCI, mild cognitive impairment; SD, standard deviation. *Note*: Simple slopes were calculated to facilitate interpretation of effect modification. The figures present regression lines on the association between the air pollution mixture and MoCA score (A) or suspected mild cognitive impairment (B) at average rate (black line), high (+1 SD, dark gray line), and low (−1 SD, light gray line) levels of the composite cognitive reserve (contributors). All models were adjusted for age, sex, body mass index, smoking status, alcohol consumption, diabetes, and hypertension.

The composite cognitive reserve (contributors) significantly moderated the relationship between the air pollution mixture and MoCA score (Table 2 and Figure 3A). In individuals with an average level of cognitive reserve, a 1–SD increase in the air pollution mixture was associated with a 0.24-point decrease in MoCA scores (95% CI: −0.311 to −0.163). This association was attenuated in individuals with higher cognitive reserve (*β* = −0.116; 95% CI: −0.253 to 0.021) and intensified in those with lower cognitive reserve (*β* = −0.362; 95% CI: −0.369 to −0.347). Among individual components of cognitive reserve, education level also significantly moderated the association between the air pollution mixture and MoCA score. Occupation, social engagement, and brain reserve did not significantly moderate the association between the air pollution mixture and MoCA score.

**Table 2.** Moderating effects of cognitive reserve contributors and brain reserve on the associations between air pollution mixture and MoCA score

Moderators	All participants (*n* = 650)			
	Beta (*β*)	95% CI		*P*-value^b^
		Lower	Upper	
**Cognitive reserve contributors**				
*Composite cognitive reserve (contributors)*				
Air pollution mixture	−0.239	−0.311	−0.163	<0.001
Cognitive reserve contributors	0.444	0.374	0.514	<0.001
Air pollution mixture × composite cognitive reserve (contributors)	0.123	0.058	0.184	<0.001
*Education levels*				
Air pollution mixture	−0.239	−0.308	−0.163	<0.001
Education levels	0.446	0.378	0.515	<0.001
Air pollution mixture × education levels	0.120	0.053	0.181	<0.001
*Occupation*				
Air pollution mixture	−0.119	−0.187	−0.038	0.002
Occupation	0.111	0.051	0.168	<0.001
Air pollution mixture × occupation	0.056	−0.003	0.111	0.052
*Social engagement*				
Air pollution mixture	−0.172	−0.304	−0.027	0.014
Social engagement	0.349	0.161	0.487	<0.001
Air pollution mixture × social engagement	0.105	−0.065	0.257	0.201
**Brain reserve^a^**				
Air pollution mixture	−0.089	−0.157	−0.009	0.020
Brain reserve	−0.178	−0.265	−0.100	<0.001
Air pollution mixture × brain reserve	−0.040	−0.107	0.043	0.294

*Note*: The air pollution mixture included PM_***10***_, PM_***2.5***_, and NO_***2***_. All models were adjusted for age, sex, body mass index, smoking status, alcohol consumption, diabetes, and hypertension.

*Abbreviations:* CI, confidence interval; MoCA, Montreal Cognitive Assessment.

a Brain reserve was quantified using the ventricle-to-brain ratio, calculated as cerebrospinal fluid divided by the sum of total gray matter volume and total cerebral white matter, multiplied by 100. To normalize the distribution, brain reserve values were log-transformed.

b Two-sided *P*-values below 0.05 were considered statistically significant.

### Moderating effects of cognitive reserve contributors and brain reserve on air pollution-associated MCI

The composite cognitive reserve (contributors) significantly moderated the relationship between the air pollution mixture and suspected MCI (Table 3 and Figure 3B). In individuals with an average level of cognitive reserve, a 1–SD increase in the air pollution mixture was associated with a 0.4% (*β* = 0.041; 95% CI: 0.017 to 0.065) increased probability of suspected MCI. This association was augmented in individuals with low cognitive reserve (*β* = 0.097; 95% CI: 0.095 to 0.099) and attenuated in individuals with high cognitive reserve (*β* = −0.015; 95% CI: −0.060 to 0.030). Each component of cognitive reserve significantly moderated the association between the air pollution mixture and suspected MCI. By contrast, brain reserve did not significantly moderate the association between the air pollution mixture and suspected MCI.

**Table 3.** Moderating effects of cognitive reserve contributors and brain reserve on the associations between air pollution mixture and suspected mild cognitive impairment

Moderators	All participants (*n* = 650)			
	Beta (*β*)	95% CI		*P*-value^b^
		Lower	Upper	
**Cognitive reserve contributors**				
*Composite cognitive reserve (contributors)*				
Air pollution mixture	0.041	0.017	0.065	0.001
Cognitive reserve contributors	−0.117	−0.146	−0.088	<0.001
Air pollution mixture × composite cognitive reserve (contributors)	−0.056	−0.078	−0.034	<0.001
*Education levels*				
Air pollution mixture	0.047	0.019	0.068	<0.001
Education levels	−0.121	−0.151	−0.092	<0.001
Air pollution mixture × education levels	−0.052	−0.075	−0.021	<0.001
*Occupation*				
Air pollution mixture	0.023	−0.007	0.044	0.083
Occupation	−0.029	−0.045	−0.009	<0.001
Air pollution mixture × occupation	−0.032	−0.052	−0.015	<0.001
*Social engagement*				
Air pollution mixture	0.087	0.021	0.153	0.012
Social engagement	−0.127	−0.186	−0.054	<0.001
Air pollution mixture × social engagement	−0.086	−0.150	−0.021	0.009
**Brain reserve^a^**				
Air pollution mixture	0.022	−0.012	0.045	0.124
Brain reserve	0.052	0.020	0.086	0.002
Air pollution mixture × brain reserve	0.024	−0.006	0.049	0.091

*Note*: The air pollution mixture included PM_***10***_, PM_***2.5***_, and NO_***2***_. All models were adjusted for age, sex, body mass index, smoking status, alcohol consumption, diabetes, and hypertension.

Abbreviations: CI, confidence interval; MCI, mild cognitive impairment.

a Brain reserve was quantified using the ventricle-to-brain ratio, calculated as cerebrospinal fluid divided by the sum of total gray matter volume and total cerebral white matter, multiplied by 100. To normalize the distribution, brain reserve values were log-transformed.

b Two-sided *P*-values below 0.05 were considered statistically significant.

## Discussion

This study is, to the best of our knowledge, the first to investigate the moderating effects of cognitive and brain reserves on the associations between the air pollution mixture and cognitive function in dementia-free older adults. This study advances existing knowledge by integrating both cognitive and brain reserve frameworks within a single analytical model while also modeling air pollution as a latent mixture (PM_10_, PM_2.5_, and NO_2_) to better reflect real-world co-exposures. This integrative approach provides a multidimensional perspective that extends beyond prior research, which typically examined single pollutants or individual reserve factors separately. We found that the associations of the air pollution mixture with the MoCA score and risk of suspected MCI were significantly moderated by the composite cognitive reserve (contributors) but not by brain reserve in dementia-free adults. Specifically, the strength of the association between the air pollution mixture and MoCA score was −0.24 in individuals with an average level of composite cognitive reserve (contributors). However, this association was attenuated to −0.12 at higher levels and intensified to −0.36 at lower levels of composite cognitive reserve (contributors).

Numerous epidemiological studies have consistently demonstrated the moderating effects of individual cognitive reserve components, such as education and social engagement, on the association between air pollution and cognitive function. Individuals with higher education levels were less affected by air pollution-related cognitive impairment, highlighting the protective role of education in reducing the neurotoxic effects of air pollution (Duchesne et al., 2022; Kulick et al., 2020; Wang et al., 2020). However, the findings on social engagement were varied (Frndak et al., 2024; Ilango et al., 2023; Tallon et al., 2017; Tan et al., 2021). While two large population-based studies found a significant moderating effect of social engagement on the association between air pollution and cognitive impairment (Frndak et al., 2024; Tan et al., 2021), other studies reported no such effect (Ilango et al., 2023; Tallon et al., 2017). Specifically, a study of cognitively typical individuals found no moderating effect of social engagement, such as involvement in clubs, religious services, or concerts (Ilango et al., 2023), while another study showed no moderating effect when social engagement was defined by the frequency of interactions with friends and family (Tallon et al., 2017). This discrepancy may be attributed to the definitions of social engagement, quality, and frequency of social interactions in the studied activities, and variations in cultural and contextual factors. In the present study, we constructed composite cognitive reserve (contributors) by combining education, occupation, and social engagement, as composite contributors are known to be more strongly associated with better cognitive function in older adults than any single contributor (Khalaila et al., 2024; Yang et al., 2024). Although the moderating role of occupation has not been investigated in the context of air pollution-related cognitive impairment, a recent meta-analysis found that individuals with more cognitively demanding jobs were 44% less likely to develop MCI (Huang et al., 2020). By incorporating occupation into the composite cognitive reserve (contributors), our findings demonstrated that a higher composite of cognitive reserve moderates the effect of air pollution on cognitive decline. This suggests the importance of considering multiple aspects of cognitive reserve to fully understand its protective potential.

Several possible mechanisms may underlie the moderating effects of cognitive reserve on the association between air pollution and cognitive impairment. It has been established in animal studies that long-term exposure to air pollution (i.e., PM_2.5_) induces greater amyloid plaque deposition and higher hyperphosphorylated tau levels (the key mechanisms of Alzheimer’s disease) (Patten et al., 2021; Sahu et al., 2021). Furthermore, an epidemiological study showed that cognitive reserve contributors (e.g. education) were associated with a slower rate of CSF beta-amyloid 42 accumulation (Lo & Jagust, 2013), suggesting that cognitive reserve contributors may mitigate air pollution–related cognitive decline by slowing amyloid deposition in the brain. In addition to amyloid and tau pathology, air pollution exposure has been consistently linked to neuroinflammation, oxidative stress, and disruption of the cerebrovascular unit, including blood–brain barrier dysfunction (Calderón-Garcidueñas et al., 2004). These processes may impair neuronal signaling, synaptic integrity, and large-scale brain network efficiency, thereby contributing to cognitive decline. Within this neurobiological context, cognitive reserve may confer resilience not by preventing the accumulation of pathology itself, but by modifying how such pathology is functionally expressed. Individuals with higher cognitive reserve may be better able to utilize more efficient neural networks, recruit alternative cognitive strategies, or maintain compensatory neuroplastic responses in the presence of pollution-related neurotoxic insults (Weiler et al., 2018). An alternative mechanism for the moderating effects of cognitive reserve is synaptic plasticity. A study of cognitively typical older adults found that those with higher cognitive reserve maintained better cognitive function, even among individuals with similar beta-amyloid pathology (i.e., low plasma beta-amyloid 42/40 ratios) (Yaffe et al., 2011). Cognitive reserve theory suggests that individuals with higher levels of brain activity (e.g. higher education level or exposure to cultural, social, physical, and cognitive activities) develop increased neuronal signaling, neurogenesis, and synaptic plasticity, leading to more complex and efficient neural networks (Stern, 2012). This may enable individuals to better compensate for pathological changes, potentially allowing them to remain asymptomatic for longer periods (Stern, 2012).

In the present study, cognitive reserve showed a significant moderating effect on the association between the air pollution mixture and cognitive function. Cognitive reserve reflects a broader set of lifelong experiences and activities, which contribute to the brain’s functional capacity to adapt and compensate for damage (Barulli & Stern, 2013; Stern, 2012; Stern et al., 2020). In addition, cognitive reserve contributors, such as education, occupation, and social engagement, may also capture broader social and health-related factors. Educational attainment and occupational status are associated with chronic stress, discrimination, and overall physical health, all of which may independently affect cognitive function (Crielaard et al., 2021; Seitz & Steger, 2025). Hence, it is possible that the observed moderation by cognitive reserve contributors at least in part reflected these intertwined psychosocial and health pathways rather than cognitive capacity alone. Given this integrated biological and social perspective of cognitive reserve, we believe that enhancing cognitive reserve may mitigate the cognitive impact of air pollution exposures. Among the studied cognitive reserve contributors, early life factors, such as educational attainment or occupational complexity, may have limited applicability for interventions, particularly in the elderly. Instead, late-life factors, such as social activities, may be promising modifiable factors for the prevention of cognitive impairment in this population. Indeed, a recent meta-analysis found that individuals with poor social relationships had a 15% higher risk of cognitive decline than those with strong social connections (Piolatto et al., 2022). Focusing on modifiable components of cognitive reserve, such as social engagement, may be effective in reducing the risk of cognitive impairment attributable to air pollution exposure.

It is noteworthy that brain reserve did not moderate the effect of air pollution exposures on cognitive function. The absence of the moderating effect of brain reserve may partly reflect fundamental differences in how structural and functional reserves operate. Whereas cognitive reserve represents functional adaptive capacity shaped by lifelong experiences, brain reserve reflects relatively stable anatomical attributes such as brain volume and neuronal density (Stern et al., 2020). Another possible explanation is that the VBR represents a global structural measure, whereas air pollution may exert more regionally specific effects on brain areas critical for cognition, such as the hippocampus and prefrontal cortex (Cho et al., 2020; Gale et al., 2020). These localized structural changes may not be adequately captured by a single whole-brain metric, potentially limiting the sensitivity of brain reserve to detect effect modification in this context. Consequently, global measures of structural reserve, such as the VBR, may be insufficient to capture short-term or regionally specific neuroadaptive responses to air pollution. Furthermore, the VBR had a median value of 0.12 (25%–75%, 0.10–0.14). This finding indicates a relatively narrow distribution of structural brain reserve. This limited variability may be attributable to characteristics of the study population. The sample consisted primarily of healthy older adults without major neurological conditions such as dementia or stroke. Future studies using region-specific structural measures, such as hippocampal volume or cortical thickness in frontal networks, together with cognitive reserve, may help clarify how structural vulnerability and functional reserve jointly influence susceptibility to air pollution–related cognitive impairment.

This study had several limitations. First, our findings may not be generalizable to other populations. Although studies from various countries have demonstrated the moderating role of cognitive reserve in air pollution-related cognitive impairment, the definition of social engagement (a key factor of cognitive reserve) may vary across cultures. Since social engagement holds different meanings across cultural contexts (Rosselli, Uribe, Ahne, & Shihadeh, 2022), replication in diverse racial and ethnic groups is needed to validate these results. Second, there is the possibility of exposure misclassification. In this study, individual long-term exposures to air pollution were assessed as 5-year average concentrations based on residential addresses; this method may not fully capture individual-level variations in exposure, such as mobility, indoor air quality, and local topography. Given that our participants were elderly and may have had reduced mobility, residential exposure was likely to provide a reasonable estimate of overall exposure. To improve accuracy, future research should incorporate data on personal air pollution monitoring, individuals’ time-activity patterns, and indoor air quality data (Park, Lee, & Jung, 2022). Third, we did not consider the quality or quantity of social engagement. Specifically, social engagement was measured using a binary format (yes/no) for each activity. However, by collecting information across multiple domains – religious activities, social gatherings, sports or gymnasium involvement, and volunteer work – and combining these into a latent variable, we provided a more comprehensive representation of the participants’ social engagement and identified its potential moderating effects. Future studies may adopt more detailed frameworks that quantify participation across intellectual, physical, and social domains, as demonstrated in prior work (Scarmeas et al., 2001). Fourth, the PM_2.5_ exposure estimates were derived from nationwide monitoring data available only in 2015, and therefore, the 1-year average concentration in 2015 was used to estimate exposure. Thus, there is a temporal mismatch between PM_2.5_ estimates and the cognitive assessment data for participants scanned in 2014 (*n* = 88). The resulting exposure misclassification may be non-differential because exposure assessment was independent of outcome ascertainment. Future studies with longitudinally aligned air pollution and cognitive assessments are warranted to confirm these findings. Finally, we defined “suspected MCI” using the MoCA screening tool and applied a cutoff score of ≤19 (Lee et al., 2008). Although this approach minimizes the risk of overestimation, it may exclude individuals with MCI. Nevertheless, we observed a prevalence of suspected MCI (15%) that was consistent with the average prevalence reported in large-scale meta-analyses of older adults aged ≥50 years (16%) (Bai et al., 2022).

In conclusion, we showed that cognitive reserve moderated the effect of air pollution on cognitive function in dementia-free adults. The effect of the air pollution mixture on cognitive function was attenuated in individuals with higher cognitive reserve. These findings highlight the importance of functional resilience, shaped by life-course experiences, in buffering the adverse cognitive effects of environmental neurotoxic exposures. From a public health perspective, our results further suggest that cognitive reserve contributors represent potentially modifiable factors that may reduce vulnerability to air pollution–related cognitive impairment. Because key contributors, such as education and social engagement, can be supported or enhanced even later in life, community-based interventions that promote cognitively stimulating activities and social participation may offer feasible strategies to mitigate neurocognitive risks associated with air pollution exposure, particularly in aging populations.

## Supporting information

10.1017/S0033291726103407.sm001 Ko et al. supplementary material Ko et al. supplementary material

## Supplementary material

The supplementary material for this article can be found at http://doi.org/10.1017/S0033291726103407.
