# Supplementary material for: Cognitive reserve and effects of air pollution mixture on cognitive function in dementia-free adults
Source: Psychol Med. 2026 Feb 2;56:e36. doi: 10.1017/S0033291726103407 (PMC12902438; doi:10.1017/S0033291726103407)
Supplement: Ko et al. supplementary material [file S0033291726103407sup001.docx]

**Supplementary Table 1. PLS-SEM model evaluation results (validity)**

| Model (A): MoCA score | | |
| --- | --- | --- |
| Evaluation indicator | | Evaluation |
| Indicator validity | |  |
|  | Proportion of total variance explained, Adjusted R-squared | Composite cognitive reserve (contributors): 0.43  Brain reserve: 0.13 |
|  | Multicollinearity, VIF (<5.0) | Composite cognitive reserve (contributors): 1.10  Brain reserve: 1.04 |
| Model (B): Suspected mild cognitive impairment | | |
| Evaluation indicator | | Evaluation |
| Indicator validity | |  |
|  | Proportion of total variance explained, Adjusted R-squared | Composite cognitive reserve (contributors): 0.24  Brain reserve: 0.13 |
|  | Multicollinearity, VIF (<5.0) | Composite cognitive reserve (contributors): 1.13  Brain reserve: 1.05 |

*Abbreviations:* MoCA, Montreal Cognitive Assessment; PLS-SEM, partial least squares structural equation modeling; VIF, variance inflation factor.

*Notes:* The composite cognitive reserve (contributors) was derived from educational level, occupation, and social engagement. The R^2^ values of the endogenous constructs were used to evaluate the model fit and indicate how well the data points fit the line. The R^2^ values were categorized as small (0.02 ≤  R^2^  < 0.13), medium (0.13 ≤ R^2^  < 0.26), and large (0.26 ≤ R^2^). Additionally, the VIF was used to assess multicollinearity among formative model variables.
